# Mosquitoes established in Lhasa city, Tibet, China

**DOI:** 10.1186/1756-3305-6-224

**Published:** 2013-08-06

**Authors:** Qiyong Liu, Xiaobo Liu, Cirendunzhu, Alistair Woodward, Pengcuociren, Li Bai, Baimaciwang, Shaowei Sang, Dazhen, Fangjun Wan, Lin Zhou, Yuhong Guo, Haixia Wu, Guichang Li, Liang Lu, Jun Wang, Dawa, Cordia Chu, Xiraoruodeng

**Affiliations:** 1 State Key Laboratory for Infectious Diseases Prevention and Control, National Institute for Communicable Disease Control and Prevention, Chinese Center for Disease Control and Prevention, 155 Changbai Road, Changping District, Beijing 102206, P. R. China; 2 WHO Collaborating Centre for Vector Surveillance and Management, 155 Changbai Road, Changping District, Beijing 102206, P. R. China; 3 China CDC Key Laboratory of Surveillance and Early-Warning on Infectious Disease, 155 Changbai Road, Changping District, Beijing 102206, P. R. China; 4 Tibet Center for Disease Control and Prevention, 21 Linkuo North Road, Lhasa, 850000, Tibet, P. R. China; 5 School of Population Health, University of Auckland, Auckland, New Zealand, Private Bag 92019, Auckland 1142, New Zealand; 6 Centre for Environment and Population Health, Nathan Campus, Griffith University, 170 Kessels Road, Queensland 4111, Nathan QLD, Australia

**Keywords:** Mosquitoes, *Culex pipiens* complex, Multiplex PCR, Established, Lhasa

## Abstract

**Background:**

In 2009, residents of Lhasa city, Tibet Autonomous Region (TAR), China reported large numbers of mosquitoes and bites from these insects. It is unclear whether this was a new phenomenon, which species were involved, and whether these mosquitoes had established themselves in the local circumstances.

**Methods:**

The present study was undertaken in six urban sites of Chengguan district Lhasa city, Tibet. Adult mosquitoes were collected by bed net trap, labor hour method and light trap in August 2009 and August 2012. The trapped adult mosquitoes were initially counted and identified according to morphological criteria, and a proportion of mosquitoes were examined more closely using a multiplex PCR assay.

**Results:**

907 mosquitoes of the *Culex pipiens* complex were collected in this study. Among them, 595 were females and 312 were males. There was no significant difference in mosquito density monitored by bed net trap and labor hour method in 2009 and 2012. Of 105 mosquitoes identified by multiplex PCR, 36 were pure mosquitoes (34.29%) while 69 were hybrids (65.71%). The same subspecies of *Culex pipiens* complex were observed by bed net trap, labor hour method and light trap in 2009 and 2012.

**Conclusion:**

The local *Culex pipiens* complex comprises the subspecies *Cx. pipiens pipiens, Cx. pipiens pallens*, *Cx. pipiens quinquefasciatus* and its hybrids. Mosquitoes in the *Cx. pipiens* complex*,* known to be, potentially, vectors of periodic filariasis and encephalitis, are now present from one season to the next, and appear to be established in Lhasa City, TAR.

## Background

Once established in high altitude regions, some mosquito species may threaten the health of humans and vertebrates due to their ability to transmit numerous diseases [1-5]. With a permanent resident population of 559, 423 at the 2010 Chinese Census, Lhasa city, which is the administrative capital of the Tibet Autonomous Region (TAR), China is situated on the northern bank of the Lhasa River, a tributary of the Yarlung Zangbo, in the mid-south of TAR. To the east and southeast of Lhasa are the regions of Nyingchi and Sharman; Nagqu neighbours Lhasa on the north and west; Xigaze lies on its southwest. Among a total of 29,518 square kilometers, the urban area of Lhasa is just 50 square kilometers. Standing on a plain over 3,650 meters (13,000 feet) above sea level and surrounded by towering mountains, Lhasa is known as the “city of the sun.” With an annual average temperature of 7.5°C, its average temperature in January is 2.3°C and 15.4°C in July. The climate here is of the temperate plateau monsoon type. Lhasa has an annual precipitation of 426 millimeters with rain falling mainly in July, August and September.

The subspecies in the *Culex pipiens* complex, may transmit a range of pathogens including the West Nile [6-9] and St. Louis encephalitis viruses [10], avian malaria, and filarial worms [11-13]. Members of the *Cx. pipiens* complex includes *Cx. pipiens*, *Cx. quinquefasciatus*, *Cx. australicus*, and *Cx. globocoxitus*[11]. *Cx. pipiens* has two recognized subspecies: *Cx. pipiens pipiens* and *Cx. pipiens pallens*. *Cx. pipiens pipiens* is an Old World taxa originally distributed from Northern Europe to the highlands of South Africa. *Cx. pipiens pallens* is found east of the Urals across temperate Asia [14]. *Cx. quinquefasciatus* typically thrives in tropical and sub-tropical regions, including the African lowlands, Americas, Asia, and Australia [15]. As to ecological preference, *Cx. pipiens* (*Cx. pipiens pipiens* &*Cx. pipiens pallens*) and *Cx. pipiens quinquefasciatus*[15] are found in most inhabited areas globally and are often closely associated with humans, earning them the names of northern and southern house mosquitoes, respectively [11]. In addition, *Cx. p. pipiens*also has two recognized forms “pipiens” and “molestus”, which differ dramatically in ecology. Though several members of the complex have limited geographic distributions [12]. *Cx. pipiens* (*Cx. pipiens pipiens* &*Cx. pipiens pallens*) and *Cx. quinquefasciatus* can hybridize extensively when their ranges overlap. Extensive introgression exists between populations of *Cx. pipiens* and *Cx. quinquefasciatus* in North America, Argentina, Madagascar, Japan and the Republic of South Korea [16-19]. *Cx. australicus* and *Cx. globocoxitus* are restricted to Australia [12].

In China, *Cx. pipiens* complex consists of four subspecies [20], including *Cx. pipiens pipiens*, *Cx. pipiens quinquefasciatus*, *Cx. pipiens pallens* and *Cx. pipiens molestus*. *Cx. pipiens quinquefasciatus* cannot be considered as a separate species and *Cx. pipiens pallens* is not an intermediate form. *Cx. pipiens molestus* is present in the underground water system in Beijing and Shenyang, China [21]. The usual altitude of *Cx. pipiens pipiens* is lower than 3,000 m. In eastern Colorado, *Cx. pipiens pipiens* activity occurs primarily in the populated valleys at lower elevation, diminishing rapidly at higher levels (>3,000 m) [22]. Alvaro Diaz-Badillo *et al.* reported that *Cx. pipiens pipiens*, *Cx. pipiens quinquefasciatus*, and their hybrids were all present in Mexico City (2,200 m) [7]. In China, *Cx. pipiens pipiens* has been identified in Xinjiang Uygur Autonomous Region. *Cx. pipiens quinquefasciatus* occurred in areas south of 32°N. Xiaohong Sun *et al.* collected seventy-five *Cx. pipiens quinquefasciatus* in northeastern Yunnan Province (2,500-3,000 m) during 2005 and 2006 [23]. *Cx. pipiens pallens* distributed in areas north of the Yangtse River [24]. The highest elevation at which *Cx. pipiens pallens* has been observed in China is 2,900 metres, in Mainling County, Nyingchi area, Tibet [25].

Identifying members of the *Cx. pipiens* complex and other sibling species by morphologic methods is time-consuming and restricted to adult males [12,26]. Other techniques, such as allozyme analyses [17], restriction fragment length polymorphism (RFLP) analysis of PCR products [27], only distinguish between the two major taxa of the complex: *Cx. pipiens* and *Cx. quinquefasciatus*. To solve this problem, Smith and Fonseca developed assays that use polymorphisms in the second intron of the acetylcholinesterase-2 (*ace-2*) locus to identify members of the *Cx. pipiens* complex and other sibling species. The same method may be used to detect introgression between *Cx. pipiens* and *Cx. quinquefasciatus*[12,27,28]. Extensive population level examination of most of the species shows they consistently generate unique fragments that may be easily resolved by electrophoresis on agarose gels. This method permits the rapid and reliable identification of local mosquitoes.

In recent years, there have been numerous changes that might assist mosquitoes to reach Lhasa and become established there. These include: global warming [29-35], increasing international trade and tourism, population growth and mobility [36], transport improvements (such as completion of the Qinghai-Tibet Railway in 2006, the Qinghai-Tibet Highway, Sichuan-Tibet Highway and China-Nepal International Road, and the construction of the Gonggar Airport) [37], changing rainfall patterns [38], and developments in agriculture, urbanization and industrialization [39]. There are no official records to show whether mosquitoes existed in Lhasa city before 2009. In 2009, reports appeared in public media concerning the emergence of mosquitoes in Lhasa city. In addition, approximately 85.3 percent of local respondents said they were bitten by mosquitoes from the beginning of 2009 to the end of 2012, and almost one in 20 (4.5%) had to attend hospital for treatment for severe inflammation and local complications (Qiyong Liu *et al*., unpublished questionnaire survey in Lhasa in 2012). Therefore, this phenomenon is already perceived to be a serious public health problem. However, it is unclear which species of mosquitoes were involved, and whether these mosquitoes have indeed established themselves locally. This study was undertaken to test the media reports and to determine whether mosquitoes are now established in the city. The results provide the first scientific assessment of mosquitoes in Lhasa and provide a foundation for development of measures to control mosquito-borne diseases in Lhasa in the future.

## Methods

### Study sites

**Figure 1:**
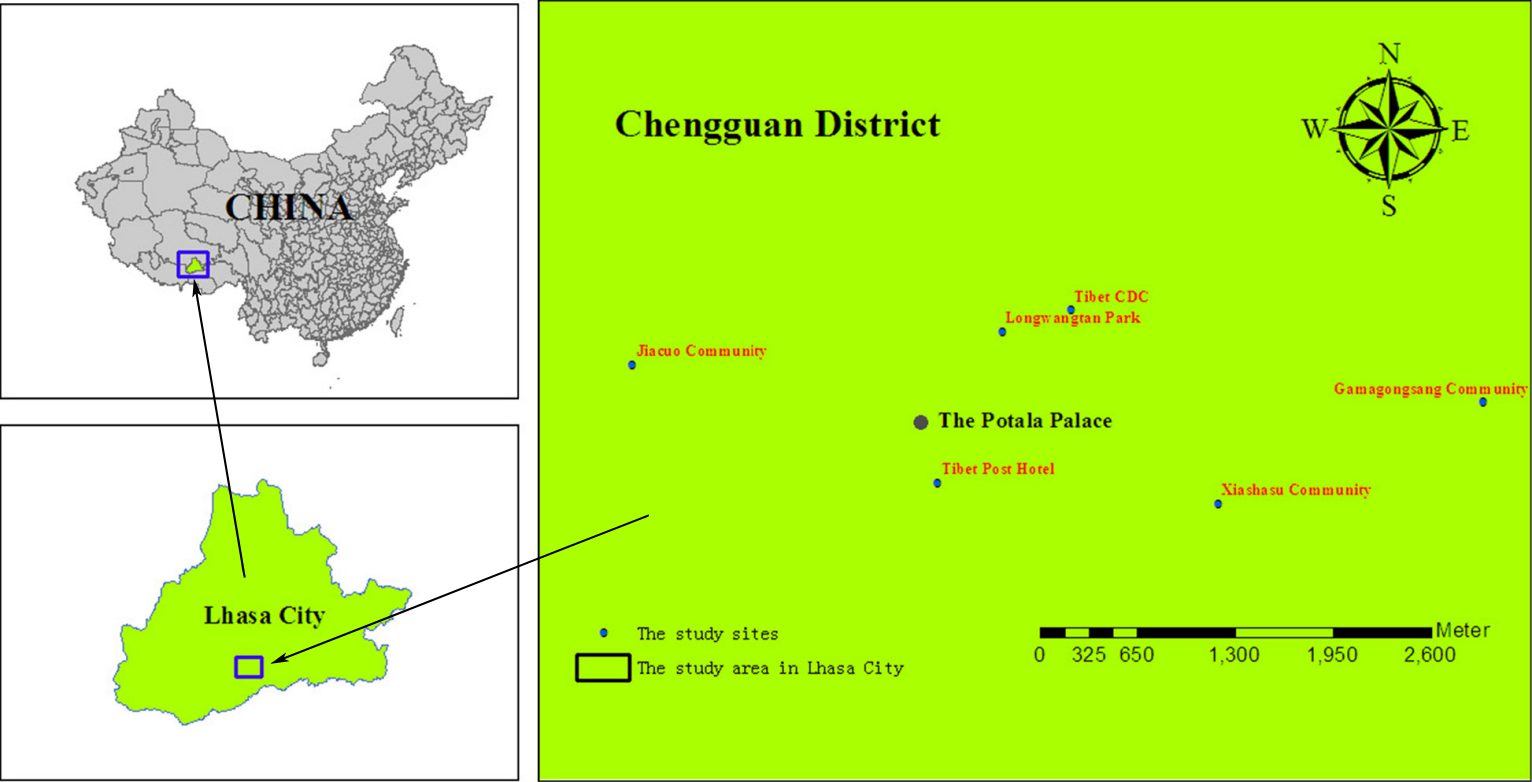
**The research sites of this study.** These sites include Tibet Center for Disease Control and Prevention (Tibet CDC) (N29°39.682’, E91°07.433’), Longwangtan Park (N29°39.582′, E91°07.151′), Tibet Post Hotel (N29°39.164′, E91°07.398′), Gamagongsang Community (N29°29.307′, E91°08.872′), Xiashasu Community (N29°39.284′, E91°07.867′) and Jiacuo Community (N29°39.429′, E91°05.375′).

The present study was undertaken in six urban sites of Chengguan district during August 2009 and August 2012. The sites were selected to be broadly representative of the geographic conditions and socio-economic characteristics of urban Lhasa. They included Tibet Center for Disease Control and Prevention (Tibet CDC), Longwangtan Park, Tibet Post Hotel, Gamagongsang Community, Xiashasu Community and Jiacuo Community (Figure 1).

Tibet CDC lies to the northeast of the Potala Palace. The campus includes many family dormitory buildings and well-established trees (cypresses). Leaks from water pipes and the irrigation of lawns provide potential breeding sites for mosquitoes. Longwangtan Park lies to the northeast of the Potala Palace and features dense vegetation and a lake, with many fish and water birds. Tibet Post Hotel lies to the southeast of the Potala Palace, close to Longwangtan Park, it has many cypresses in its courtyard. Gamagongsang Community lies to the east of the Potala Palace with a population of 2,149 people within 837 households. There is limited infrastructure such as drainage systems and roads, and there are no parks or other urban green spaces. Xiashasu Community lies to the southeast of the Potala Palace with a population of 1,519 people within 907 households. Because of the famous “Dazhao Temple”, this community is the most crowded in urban Lhasa. Residents tend to be less educated, with lower incomes, restricted living spaces and poor dwelling conditions. Jiacuo Community lies to the northwest of the Potala Palace and adjacent to a large park. Residents are relatively wealthy and mainly live in self-built single family houses with small yards.

### Mosquito collection and initial morphological identification

In this study, outdoor bed net traps (Tibet CDC, Longwangtan Park), light trap collection (Tibet Post Hotel, Tibet CDC, Gamagongsang Community, Xiashasu Community and Jiacuo Community) and labor hour method (Outpatient building and Residential area in Tibet CDC) were employed to collect adult mosquitoes in August 2009 and August 2012. Studies were carried out on the same days of the month, three years apart. The surveillance method was the standard method released by the General Administration of Quality Supervision, Inspection and Quarantine of the People’s Republic of China and Standardization Administration of the People’s Republic of China (surveillance methods for vector density-mosquito, GB/T 23797–2009).

The bed net traps were applied between 19:00 and 24:00 (the peak time for mosquitoes), taking account of the time for sunset in Lhasa city (generally 20:00 in August). Bed net traps were placed close to potential breeding habitats, at intervals of 100 m. The distance from the bed net traps to the nearest resident’s house was also about 100 m. The size of bed net traps was 1.5 m × 1.2 m × 1.5 m, with twenty-five centimeters between the floor and the bottom of the bed net traps. Some members of staff at Tibet CDC and China CDC were selected as human baits. These members (under double bed net traps to avoid mosquitoes bites) were also used repeatedly throughout the entire duration of the study [40]. Every hour, all mosquitoes inside the bed net traps were collected by an electrical aspirator for 15 minutes per hour throughout the 5 hour period. In 2009, the bed net traps were carried out on Aug.3rd - 4th in Tibet CDC (Lawn) (total of 3 bed net traps) and Aug.3rd in Longwangtan Park (total of 3 bed net traps). In 2012, the bed net traps were carried out on Aug. 7th - 8th in Tibet CDC (Lawn) (total of 4 bed net traps) and Aug. 10th - 11th in Longwangtan Park (total of 4 bed net traps).

An electric aspirator was employed for 15 minutes to collect mosquitoes inside an outpatient building and the residential area of Tibet CDC. In 2009, the labor hour method was carried out on Aug.4th in Tibet CDC (outpatient building) (total of 1 person) and Aug.3rd in Tibet CDC (residential area) (total of 1 person). In 2012, the labor hour method was carried out on Aug.8th in Tibet CDC (outpatient building) (total of 1 person) and Aug.7th in Tibet CDC (residential area) (total of 1 person).

Kung Fu Xiaoshuai miniature light traps (Photocatalytic Miewen Ying supply device; Wavelength: 2537Å; Power: 8W; Corporation: Wuhan Environmental Protection Technology Co., Ltd. Gemstar) were used to collect adult mosquitoes. The light traps were placed in the campus of Tibet Post Hotel, Tibet CDC and Gamagongsang Community, Xiashasu Community and Jiacuo Community. Traps were hung away from interference by light sources, 1.5 m above the floor. They were turned on 1 hour before sunset (20:00) and turned off 1 hour after sunrise (08:00). In 2012, the light traps were employed from Aug.5th - 12th in Tibet Post Hotel (total of 18 light traps), on Aug.7th - 9th in Tibet CDC (Lawn) (total of 9 light traps), on Aug.10th in Gamagongsang Community (total of 2 light traps), on Aug.12th in Xiashasu Community (total of 4 light traps) and on Aug.9th in Jiacuo Community (total of 4 light traps), respectively.

Information on temperature (°C) and relative humidity (%) was obtained from http://www.weather.com.cn. During collections, ambient outdoor air temperature and relative humidity was recorded hourly using a WS-1 Thermo-Hygrometer device.

### Mosquito species identification

Each morning, the trapped adult mosquitoes were initially counted and identified according to morphological criteria using the key developed by Lu BL [24]. All collected mosquitoes were put into 1.5 ml centrifuge tubes individually and then transported to the laboratory of the Department of Vector Biology and Control in China CDC for further molecular identification. Genomic DNA was extracted from individual mosquitoes. A Qia Amp DNA Mini Kit (Qiagen Inc., CA) was adopted and DNA was extracted from the thorax of mosquitoes according to the manufacturer’s instructions.

To reveal the species composition of mosquitoes in Lhasa city, a multiplex PCR protocol was adopted using polymorphisms in the second intron of the acetylcholinesterase-2 (*ace-2*) locus, developed by Smith, J. L. & Fonseca, D. M [12]. Three forward primers (ACEquin, ACEpall and ACEpip) and one backward primer (B1246s) were adopted simultaneously. Each of the three primers was used in conjunction with the reverse primer B1246s [12,26], (Table 1). Because of limited distribution of *Cx. pipiens molestus* in China [21], the primer of *Cx. pipiens molestus* was not included in this study. Approximately 105 (14.4%) mosquitoes that were selected from four sites (two institutions and two communities) in 2012, were further identified to sub-species level.

**Table 1:** Primer sequences for the acetylcholinesterase-2 locus-based polymerase chain reaction assay

^**1**^**Primers**	**5′-3′sequences**	**Product size with B1246s (bp)**
ACEpip	5′-GGA AAC AAC GAC GTA TGT ACT-3 ′	610
ACEpall	5′-ATG GTG GAG ACG CAT GAC G-3′	478
ACEquin	5′-CCT TCT TGA ATG GCT GTG GCA-3 ′	274
B1246s	5′-TGG AGC CTC CTC TTC ACG G-3 ′	

Primer sequences for the acetylcholinesterase-2 (*ace-2*) locus-based polymerase chain reaction assay.

^1^Primers of this multiplex PCR assay were designed based on the sequence differences in the acetylcholinesterase-2 (*ace-2*), developed by Smith, J. L. & Fonseca, D. M.

The PCR assay was optimized for 25 ul volumes. Reactions contained 10 × PCR buffer, 250 uM of each dNTP, one unit of Taq polymerase, and genomic DNA. The amplification program consisted of one cycle at 94°C for five minutes, followed by 35 cycles at 94°C for 30 seconds, 55°C for 30 seconds, 72°C for one minute, and one cycle at 72°C for five minutes.

In addition, to further verify the subspecies of the *Cx. pipiens* complex, further sequence analysis of the *Ace-2* gene for both some pure *Cx. pipiens pipiens*, *Cx. pipiens quinquefasciatus*, *Cx. pipiens pallens* and possible hybrids among them were conducted by Tsingke Company (Beijing, China) using the same mosquitoes as the multiplex PCR assay. Approximately three each of pure and possible hybrid mosquitoes were further sequenced in this study.

### Statistical analysis

Information was recorded on the date of the collections, number of bed net traps, number of light traps, duration of mosquito catch (h), the presence and gender of *Cx. pipiens* complex mosquitoes. An independent-sample T test was adopted to compare the density of mosquitoes between 2009 and 2012 after a satisfactory check for normality of the distribution and homogeneity of variance of the data. Numbers of species identified by multiplex PCR at different sites in different years were recorded and calculated. Analysis was conducted by SPSS (Statistical Package for the Social Sciences) statistical software (version 17.0).

### Ethics statement

We obtained ethical approval from the Ethical Review Committee of Chinese Center for Disease Control and Prevention for this study (No. 201214). Permission was also obtained from the Government, the Municipal Health Bureau and Tibet CDC in the Tibet Autonomous Region.

## Results

### Morphological identification

In this study, 907 mosquitoes in total were captured including 595 female and 312 male mosquitoes (Table 2). Preliminary morphological identification demonstrated that all these mosquitoes belonged to subspecies of the *Cx. pipiens* complex [24].

**Table 2:** **The presence of the *Culex pipiens *complex in different collections during the mosquito season in Lhasa city, Tibet**

**Year**	**The study sites**	**The latitude and longitude**	**Collection method**	**Date of the collections**	**No. of bed net traps/light traps/people**	**Duration of mosquito catcher (h)**	**The presence of *Culex pipiens *complex**	**The total number of *Culex pipiens *complex**	
								♀	♂
2009	Tibet CDC (Lawn)	N29°39.682′, E91°07.433′	Bed net trap	Aug.3rd-4th	3	2.5	+	132	0
	Longwangtan Park	N29°39.582′, E91°07.151′	Bed net trap	Aug.3rd	3	4.5	+	4	1
	Tibet CDC (Outpatient building)	N 29°39.566′, E91°07.361′	Labor hour method	Aug.4th	1	0.33	+	34	0
	Tibet CDC (Residential area)	N 29°39.682′,E91°07.433′	Labor hour method	Aug.4th	1	0.33	+	4	3
2012	Tibet CDC (Lawn)	N29°39.682′, E91°07.433′	Bed net trap	Aug.7th - 8th	4	8	+	25	0
	Longwangtan Park	N29°39.582′, E91°07.151′	Bed net trap	Aug.10th - 11th	4	8	+	20	4
	Tibet CDC (Outpatient building)	N 29°39.566′,E91°07.361′	Labor hour method	Aug. 8th	1	2.00	+	0	3
	Tibet CDC (Residential area)	N 29°39.682′,E91°07.433′	Labor hour method	Aug.7th	1	1.67	+	17	9
	Tibet Post Hotel	N29°39.164′, E91°07.398′	Light trap collection	Aug.5th - 12th	18	216	+	83	58
	Tibet CDC (Lawn)	N29°39.682′, E91°07.433′	Light trap collection	Aug.7th - 9th	9	108	+	12	13
	Gamagongsang Community	N29°29.307′, E91°08.872′	Light trap collection	Aug.10th	2	24	+	2	0
	Xiashasu Community	N29°39.284′, E91°07.867′	Light trap collection	Aug.12th	4	48	+	124	53
	Jiacuo Community	N29°39.429′, E91°05.375′	Light trap collection	Aug.9th	4	48	+	135	171
Total							+	595	312

The presence of the mosquito populations in different collections during the mosquito season in Lhasa city, Tibet.

“+” means the presence of *Culex pipiens* complex. Outpatient building, residential area and lawn lies in the courtyard of Tibet CDC. All of these *Culex pipiens* complex were collected during the season of peak activity.

### Mosquitoes collected by bed net traps in different years

Using bed net traps, 132 mosquitoes (132 females) were collected in Tibet CDC (Lawn) and 5 mosquitoes (4 females, 1 male) were collected in Longwangan Park in 2009. Three years later, 25 mosquitoes (25 females) were collected in Tibet CDC (Lawn) and 24 mosquitoes (20 females, 4 males) were collected in Longwangan Park, (Table 2). The mean mosquito density was 23.72 (mosquitoes per hour per net) in 2009 while it was 3.06 (mosquitoes per hour per net) in 2012 (Table 3). There was no significant difference of mosquito density monitored by bed net traps in 2009 and 2012 (t = 1.299, df = 12, P = 0.218>0.05).

**Table 3:** **The density of the *Culex pipiens *complex in different years during the mosquito season in Lhasa city, Tibet**

**Year**	**Collection method**	**No. of bed net traps/light traps/people**	**Duration of mosquito catcher (h)**	**Mean**^**1**^	**Std. deviation**	**Std. error**	**The total number of *Culex pipiens *complex**	
							♀	♂
2009	Bed net trap	6	7	23.72	45.54	18.59	136	1
	Labor hour method	2	0.66	62.10	57.84	40.90	38	3
2012	Bed net trap	8	16	3.06	2.19	0.78	45	4
	Labor hour method	2	3.67	8.54	9.95	7.04	17	9
	Light trap collection^2^	37	444	17.59	33.16	5.45	356	295
Total	-	-	-	-	-	-	595	312

^1^Mean means mosquito per hour per trap (Bed net trap) or mosquitoes per hour per person (Labor hour method) or mosquitoes per trap per night (Light trap collection).

^2^ light trap collection only conducted in 2012.

### Mosquitoes collected by labor hour method in different years

Using the labor hour method, 34 mosquitoes (34 females) were collected in Tibet CDC (Outpatient building) and 7 mosquitoes (4 females, 3 males) were collected in Tibet CDC (Residential area) in 2009. In 2012, 26 mosquitoes (17 females, 9 males) were collected in Tibet CDC (Residential area) (Table 2). The mean mosquito density was 62.10 (mosquitoes per hour per person) and 8.54 (mosquitoes per hour per person) in 2009 and 2012, respectively (Table 3). There was no significant difference of mosquito density monitored by labor hour method in 2009 and 2012 (t = 1.291, df = 2, P = 0.326>0.05).

### Mosquitoes collected by light traps in 2012

In 2012, light traps collected 83 female and 58 male mosquitoes in Tibet Post Hotel and 12 females and 13 males in Tibet CDC (Lawn). 2 females were collected in Gamagongsang. 124 females and 53 males were collected in Xiashasu. 135 females and 171 males were collected in Jiacuo (Table 2). The mean mosquito density was 17.59 (mosquitoes per trap per night) in 2012 (Table 3).

### Multiplex PCR assay for molecular identification

**Figure 2:**
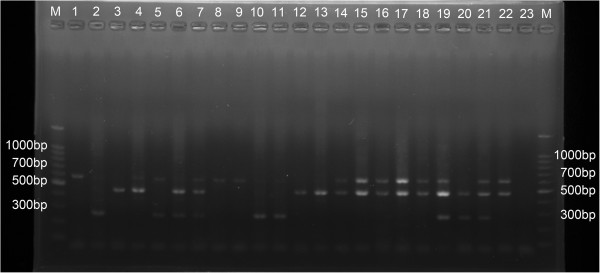
**Ethidium bromide-stained agarose gel showing multiplex PCR assay for molecular identification.** M: Maker; Positive control products from *Cx. pipiens* complex are shown in lanes1-7. Lane 1 (*Cx. pipiens pipiens* from Urumchi, Xinjiang, 610 bp); Lane 2 (*Cx. pipiens quinquefasciatus* from Dali, Yunnan, 274 bp); Lane 3 (*Cx. pipiens pallens* from Beijing, 478 bp); Lane 4 (*Cx. pipiens pipiens* &*Cx. pipiens pallens,* 1 ul ofeach DNA, 610 bp &478 bp); Lane 5 (*Cx. pipiens pipiens* &*Cx. pipiens quinquefasciatus,*1 ul of each DNA, 610 bp&274 bp); Lane 6 (*Cx. pipiens quinquefasciatus*&*Cx. pipiens pallens*, 1 ul of each DNA,274 bp&610 bp); Lane 7 (*Cx. pipiens pipiens*&*Cx. pipiens pallens*&*Cx. pipiens quinquefasciatus,* 0.67 ul of each DNA, 610 bp&274 bp&478 bp), respectively. Mosquitoes from Lhasa city and other provinces are shown in lanes 8–22, respectively. Lane 23, negative control. Outside lanes are 100 bp DNA ladders. bp = basepaires.

In this study, 105 mosquitoes from Lhasa city and another 17 mosquitoes used as positive controls from other provinces (positive control: *Cx. pipiens pipiens* from Urumchi, Xinjiang, 610 bp;*Cx. pipiens quinquefasciatus* from Dali, Yunnan, 274 bp; *Cx. pipiens pallens* from Beijing, 478 bp) were examined using the multiplex PCR assay developed by Smith, J. L. & Fonseca, D. M [12]. The results revealed that the size of the amplified product was 274 bp for *Cx. pipiens quinquefasciatus*, 478 bp for *Cx. pipiens pallens* and 610 bp for *Cx. pipiens pipiens*. Primers were successfully designed for the identification of these mosquitoes (Figure 2).

Multiplex PCR revealed that mosquitoes from Lhasa city include 36 pure mosquitoes (34.29%) and 69 hybrids (65.71%) (Table 4). The pure mosquitoes included 13 *Cx. pipiens pipiens*, 3 *Cx. pipiens quinquefasciatus* and 20 *Cx. pipiens pallens.* Possible hybrids consisted of 41 *Cx. pipiens pipiens* and *Cx. pipiens pallens*, 14 *Cx. pipiens pallens* and *Cx. pipiens quinquefasciatus*, only 1 *Cx. pipiens pipiens* and *Cx. pipiens quinquefasciatus*, and 13 among *Cx. pipiens pallens* &*Cx. pipiens quinquefasciatus* and *Cx. pipiens pipiens*.

**Table 4:** Multiplex PCR assay for molecular identification

**The study sites**	**Collection method**	**Year (number)**	**N**	**A**	**B**	**C**	**A&C**	**A&B**	**B&C**	**A&B&C**
Tibet Post Hotel (Courtyard)	Light trap collection	2012 (141);	20	6	0	0	8	0	2	4
Tibet CDC (Lawn)	Light trap collection	2012 (25);	3	0	0	3	0	0	0	0
Xiashasu Community	Light trap collection	2012 (177);	44	5	0	8	22	1	4	4
Jiacuo Community	Light trap collection	2012 (306);	38	2	3	9	11	0	8	5
Total	-	2012 (649)	105	13	3	20	41	1	14	13

N means numbers of mosquitoes.

A, B and C refers to mosquitoes of *Culex pipiens* complex. A refers to *Cx. pipiens pipiens*. B refers to *Cx. pipiens quinquefasciatus.* C refers to *Cx. pipiens pallens*.

Sequence analysis further confirmed the accuracy of multiplex PCR in this study. Based on the similarity analysis using blast, the similarity of the *Cx. pipiens quinquefasciatus* from Lhasa to the sequence of *Cx. pipiens quinquefasciatus* from central Bangladesh was 100% (273/273). The similarity of the *Cx. pipiens pallens* from Lhasa to the sequence of *Cx. pipiens pallens* from Iran was 99% (436/438). The similarity of the *Cx. pipiens pipiens* from Lhasa to the sequence of *Cx. pipiens pipiens* from Iran was 95% (530/560).

## Discussion

This is the first investigation to verify media reports of mosquitoes in Lhasa. We observed subspecies of *Cx. pipiens* complex and its hybrids on two occasions, three years apart. Our findings were based on entomological investigations in the field and multiplex PCR methods in the laboratory. In this study, there was no significant difference of mosquito density monitored by bed net trap and labor hour method in 2009 and 2012. In urban Lhasa, we observed that the ecological and geographical factors did not change significantly three years later. However, it seemed that the mean mosquito density, both using bed net traps and labor hour method, were relatively higher in 2009 than in 2012 though no statistical significance was observed. We note that the summer of 2012 did not match the high temperatures of three years earlier: the maximum temperature in 2012 was 29.0°C compared with 30.4°C in 2009 (http://www.tianqi.com). In addition, the public health campaign may also play a major role in the relatively lower density in 2012. In recent years, Lhasa accelerated the process of establishing of the National Sanitary City in China. As a key indicator, mosquito density was controlled by local health authorities and related agencies using some insecticides and similar products. This campaign might have exerted some adverse impact on the density of mosquitoes in 2012. Furthermore, with the huge development of the economy and culture in Lhasa, local citizens focus more on their health status than ever before. A variety of insecticides have been adopted to protect themselves from mosquitoes bites.

Previous studies showed that the most northerly area of *Cx. pipiens pipiens* is about 45°N in the New World and the southerly area is about 39°S, and the usual altitude of this subspecies is lower than 3,000 m [41,42]. In China, *Cx. pipiens pipiens* has been recorded only in Xinjiang Uygur Autonomous Region (northwestern China). However, previous data on the distribution of *Cx. pipiens pipiens* is limited: reliable identification depends on collection of males, which was not always the case. Furthermore, species and subspecies classification has been difficult because there were no populations of *Cx. pipiens pipiens* in Chinese laboratories. In the current study, pure *Cx. pipiens pipiens* (subspecies of *Cx. pipiens complex*) was definitively identified in urban Lhasa (an area of elevation higher than 3,600 m). This finding significantly extends present knowledge of the distribution of *Cx. pipiens pipiens* in China, and has important implications for the control of mosquitoes and mosquito-borne diseases in Lhasa city.

In Eastern Asia *Cx. pipiens pallens* transmits lymphatic filariasis and canine dogworm and may act as a vector for West Nile virus [43-46]. *Cx. p. pallens* differs from hybrids of *Cx. p. pipiens* and *Cx. quinquefasciatus*[14]. In China, *Cx. pipiens pallens* has been found north of the Yangtse River [24], but not previously at an altitude of greater than 2,900 m [25].

This study has uncovered possibly extensive hybridization among subspecies of *Cx. pipiens* complex in Lhasa city. Natural hybridization is defined as “successful matings in nature between individuals from two populations, or groups of populations, that are distinguishable on the basis of one or more heritable characters” [47]. Combinations of this kind enhance rapid evolution and may lead to speciation [48]. According to the existing literature, recurring hybridization occurs in the *Cx. pipiens* complex mostly between the two most widespread species, *Culex (Culex) pipiens* and *Cx. (Cx.) quinquefasciatus*[14]. Hybrids have been reported in North America [17,42], Argentina [19], as well as in Madagascar [49]. A multilocus genotype analysis revealed current hybridization between *Cx. p. pallens* and *Cx. quinquefasciatus* in southern Japan, Republic of Korea, and China [12,50].

In the present study, primers specifically designed for East Asia by Smith & Fonseca were adopted. These primers were successfully designed for the identification of mosquitoes in Lhasa city. In this study, positive controls were also included, in other words, *Cx. pipiens pipiens* from Urumchi, Xinjiang, *Cx. pipiens quinquefasciatus* from Dali, Yunnan, and *Cx. pipiens pallens* from Beijing. The study of the indigenous populations of mosquitoes using molecular markers allowed us to confirm the occurrence of *Cx. pipiens* complex in Lhasa city (southwest of China). Of some interest is the discovery of hybrid populations including *Cx. pipiens pipiens*, *Cx. pipiens quinquefasciatus*, *Cx. pipiens pallens* and their hybrids (65.71%) in Lhasa city.

Climate change may have played a part in the arrival of mosquitoes in Lhasa. Average temperatures increased over the Tibetan plateau from 1955 to 1996 by about 0.16°C/decade [51], much more than in China generally. From 1961 to 2000, the greatest increase in daily mean temperatures in summer (June to August) in Tibet occurred in Lhasa city [52,53]. In 2009, Tibet experienced unusually warm conditions and the maximum temperature in Lhasa reached 30.4°C, higher than the previously reported record (29.9°C in 1971). In other words, the first public reports of mosquitoes coincided with the warmest summer in Lhasa since records were first kept. It is possible that mosquitoes were introduced earlier, but numbers multiplied during the particularly hot summer of 2009. In the future, further warming is expected, and further economic development in Tibet will lead to even greater movement of freight and people. These conditions raise the risk of outbreak of mosquito-borne diseases in a population with no prior exposure to such infections [54-56]. Therefore, it is urgent to strengthen the detection and monitoring of mosquito-borne diseases in the region.

Other factors, such as demographic and environmental factors, may also play a more important role in establishing the mosquito population in Lhasa. In the last 30 years, China has undergone enormous economic growth, largely due to greatly increased international trade. This burgeoning trade has triggered environmental threats from an expanding list of biological invaders and has already caused damage to China’s environment and economy. Huge construction projects, such as the Qinghai-Tibet Railway [57], could further spread invasive mosquitoes to Lhasa city [37,58,59]. As to urbanization, Tibet entered a stage of accelerated urbanization after 1995. The large floating population from outside Tibet has become the driving force for urban expansion and the rising urbanization rate. Tibet’s urbanization rate will be up to 43% by 2020 based on a website (http://chinatibet.people.com.cn/6828539.html). At present, Lhasa is claimed to be a modern city on the “roof of the world” with a forest of new buildings and luxury hotels, restaurants and stores. Previous research revealed that urbanization serves in the formation of appropriate habitat of culicines. In Macau, recent urbanization has provided optimal habitat for the population increase in culicines [60]. In Tanzania, urbanization resulted in some changes in mosquito populations [61]. Now, Lhasa, which has a large number of tourism resources, such as the Potala Palace, Jokhang Temple, Sara Monastery, and Barkhor Street, is a popular destination for both domestic and international travelers. By April, 2008, there were over 1,600 licensed tour guides in Lhasa according to The Chinese National Tourism Administration. Tourist aircraft or trains may carry mosquitoes to urban Lhasa and subsequently threaten the health and lives of local citizens. It was reported that labor flow and travelers are significant factors contributing to the spread of dengue virus infection and chikungunya fever [62].

In summary, our investigation provides insight into the new distribution of subspecies of *Cx. pipiens* complex and its hybrids in Lhasa, Tibet. The findings mentioned above have a significant implication in public health areas, both at policy making and practical levels. The multiplex PCR assay adopted in this study will be helpful to researchers and will aid vector control programs by facilitating the rapid and reliable identification of local *Cx. pipiens* complex and its hybrids. The future focus of the control and prevention of mosquito-borne diseases in Lhasa is West Nile virus, St. Louis encephalitis viruses, avian malaria, and filarial worms. Strengthened community health education and engagement should be conducted to better guarantee the health and life safety of local citizens. The results could provide a reference for development of varieties of strategies and measures to control mosquitoes and mosquito-borne diseases at high elevation regions in the world in future.

This study has limitations since it was planned and implemented, initially, in response to public concerns, and includes information from only two time points. However, the results indicate that mosquitoes are established in a high altitude urban setting in Tibet. Further studies are needed to confirm the continuing presence of mosquitoes, to clarify the patterns of hybridization, and to shed further light on likely origins and factors influencing their distribution and establishment in Lhasa city [63].

## Conclusion

In summary, the results revealed subspecies of *Cx. pipiens* complex and its hybrids on two occasions, three years apart in urban Lhasa. There was no significant difference in mosquito density monitored by bed net trap and labor hour method in 2009 and 2012. Mosquitoes in the *Cx. pipiens* complex appear to be established in Lhasa City, TAR. Strengthened community health education and engagement should be conducted to better guarantee the health and life safety of local citizens.

## Abbreviations

TAR: Tibet autonomous region; RFLP: Restriction fragment length polymorphism; ace-2: Acetylcholinesterase-2.

## Competing interests

The authors declare that they have no competing interests.

## Authors’ contributions

QL, XL, AW and CC planned the project and wrote the paper. QL, XL, C, P, FW, B, LB, YG, D, GL, JW, SS, D and X conducted the field survey. XL, LL, L and HW carried out the multiplex PCR assay in the lab. XL, QL, CC, and AW contributed to data analysis. All authors read and approved the final manuscript.
